# Active and passive diffusion processes in complex networks

**DOI:** 10.1007/s41109-018-0100-5

**Published:** 2018-10-01

**Authors:** Letizia Milli, Giulio Rossetti, Dino Pedreschi, Fosca Giannotti

**Affiliations:** 1 University of Pisa, Largo B. Pontecorvo, 2, Pisa, Italy; 2 KDD Lab. ISTI-CNR, via G. Moruzzi, 1, Pisa, Italy

**Keywords:** Diffusion processes, Complex networks, Diffusion of information

## Abstract

Ideas, information, viruses: all of them, with their mechanisms, spread over the complex social information, viruses: all tissues described by our interpersonal relations. Usually, to simulate and understand the unfolding of such complex phenomena are used general mathematical models; these models act agnostically from the object of which they simulate the diffusion, thus considering spreading of virus, ideas and innovations alike. Indeed, such degree of abstraction makes it easier to define a standard set of tools that can be applied to heterogeneous contexts; however, it can also lead to biased, incorrect, simulation outcomes. In this work we introduce the concepts of *active* and *passive* diffusion to discriminate the degree in which individuals choice affect the overall spreading of content over a social graph. Moving from the analysis of a well-known *passive* diffusion schema, the Threshold model (that can be used to model peer-pressure related processes), we introduce two novel approaches whose aim is to provide *active* and *mixed* schemas applicable in the context of innovations/ideas diffusion simulation.

Our analysis, performed both in synthetic and real-world data, underline that the adoption of exclusively *passive*/*active* models leads to conflicting results, thus highlighting the need of *mixed* approaches to capture the real complexity of the simulated system better.

## Introduction

Information, ideas, viruses all of them have something in common: they describe different kinds of “contents” that need to be vehiculated by interacting agents to diffuse. Agents can be either individuals or animals as well as computers or other technological devices connected by a complex network describing their relations. Even if similar at a high abstraction level, diffusion process have their characteristics that profoundly affect the way they evolve. One such characteristic is undoubtedly tied to the degree of *activeness* of the agents they aimed to reach. Agents can be *passive* and doomed to suffer a diffusion process (e.g., during an outbreak of influenza) or *active* and voluntarily adopt a given behavior or idea just because they feel it right. Moreover, agents can also show both of such behaviors: in some circumstances a content can need both a certain degree of exposure of actors as well as their interest to be adopted. Indeed, such ambivalence is strictly tied to specific contents and contexts and can be modeled using different approaches.

The *activeness* distinction regards prevalently phenomena of *social contagion*, where the diffusing object is either an idea or a piece of information. *Social contagions* are often modeled using a classical approach, the Threshold model (Granovetter 1978) introduced by Granovetter in 1978. In this model the adoption of ideas or information by an individual is subject to a personal threshold; these approaches, however, tend to capture only the *passive* component of the diffusion, ignoring the user interests concerning the information. However, peer pressure is not the only component that acts as the linchpin for individual’s adoption: personal interest plays a relevant role, an *active* impulse that - once the subject is aware of the existence of the content - disregarding the peer pressure volume can inhibit/facilitate the diffusive process.

Indeed, the *active*-*passive* dichotomy have not yet been adequately addressed nor formal models considering active users in network diffusion proposed: for this reason in this study we describe variants of the threshold model aimed to start filling such gap. Moreover, in the real world exist some people that decide autonomously to adopt an idea or information without peer pressure from their friends and others that decide not to adopt that ideas. So in this work, we modeled also the spontaneous adoption phenomenon and the presence of blocked nodes.

After having characterized *active* and *passive* diffusion schema, we tackle the problem of understanding if, and how, spontaneous adoption and blocked nodes affect the diffusion of innovations/ideas. Indeed, a plethora of diffusion models can be designed to capture such behaviors - some of them even interchangeably, assigning different semantics to the variables they expose. To overcome such issue, in this work we decided to perform a simple distinction: we model *passive* approaches through deterministic diffusion rules (i.e., individual thresholds that mimic peer-pressure phenomena) and *active* ones through probabilistic ones (i.e., individual profiles that depends only on the interest of the subject into the diffusing content).

The paper is organized as follows. In Section “Related works” are introduced and discussed related works on diffusion process modeling. In Section “Social diffusion conundrum” we formalize our problem definition, characterizing the different diffusion scenarios we will analyze, namely *active*, *passive* and *mixed* diffusion. There we also introduce the algorithmic schema we used to simulate such scenarios. In Section “Experimental analysis” we approach the analytical part of our investigation: there the datasets, methodology and experimental results – for all the identified scenarios and network settings – are introduced and discussed. Finally, Section “Conclusion” concludes the paper.

## Related works

Generally, diffusion processes can be roughly broken down into three components: (i) the population on which they unfold, (ii) the mechanisms that describe their evolution, and (iii) the content of the diffusion. All those components are equally important to model, understand, simulate a diffusion process: in particular, the content spread represents the real discriminant among active/passive diffusion. Commonly, the phrase *“epidemic spreading”* is used to imply the diffusion of contagious diseases caused by biological pathogens, like influenza, measles, chickenpox as well as sexually transmitted diseases. However, a plethora of phenomena can be linked to the concept of the epidemic: examples are the spread of computer viruses (Szor 2004), as well as the spread of mobile phone virus (Havlin 2009; Wang et al. 2013), or the diffusion of knowledge, innovations, products in an online social network (Burt 1987).

Indeed, there are analogies between spreading phenomena involving different contents, the most important being their unfolding over complex networks whose nodes are characterized by their infectious state and links representing the interaction between nodes.

In this paper, we focus on a specific content of diffusion: innovations/ideas. The diffusion of innovation theory, developed by Rogers in 1962 (Rogers 2003), is one of the oldest social science theories: it aims to explain how an idea or product gains force and diffuses through a specific population or social system. The adoption of a new idea, behavior or product does not happen simultaneously in a social system; it is a process whereby some people are more suitable to adopt the innovation than others. To address the diffusion of innovation problem are often adopted variants of the *Threshold Model* (Granovetter 1978): in such model an individual has two distinct and mutually exclusive behavioral alternatives, the decision to do or not do something – i.e., adopt or not a given behavior – a decision tied on how many other people have made the same choice. Such behavior is modeled by employing individual *thresholds* to account for social pressure – e.g., a person’s threshold for adopting a behavior can be defined as the proportion of the group he would have to see adopted before he would do so.

In Watts (2002), for instance, was shown that while applying such model in a network a global diffusion cascade can occur due to the interactions between nodes and individual thresholds. However, such model presents some limitations: (i) diffusion process is ignited by a single node status perturbation, while there are many situations where multiple sources of perturbation concur to the spreading (e.g., external impulses can arrive from the mass media, advertising, friends), (ii) it does not consider the presence of individuals reluctant to adopt. When complex perturbations lie behind diffusion processes, we talk about *Complex contagion*, in which multiple sources of exposure to innovation are required before an individual adopts the change of behavior (Watts 2002; Backstrom et al. 2006; Gleeson and Cahalane 2007; Romero et al. 2011; Bakshy et al. 2012; Singh et al. 2013; Centola 2010; 2011). In such contexts, beyond the conventional threshold mechanism, recently were also investigated the effect of homophily (Aral et al. 2009; Bakshy et al. 2012; Suri and Watts 2011) and the role of external media influence (Toole et al. 2012). Conversely, the presence of reluctant individuals was addressed in Ruan et al. (2015) where was introduced a threshold-based model that includes blocked nodes as well as spontaneous adopters. The concepts of *active* and *passive* diffusion was tackled in Milli et al. (2017); in this paper was introduced two approaches whose aim is to provide active and mixed schemas applicable in the context of innovations/behaviors/ideas diffusion simulation in the static case, also introducing the concept of blocked nodes.

## Social diffusion conundrum

In this work, we tackle a particular typology of network spreading, the diffusion of innovations/behaviors/ideas: in the following, we will use such terms interchangeably and refer collectively to them as *contents* of diffusion processes.

The phrase *diffusion of innovations* is often used to describe an active process: scenarios in which, conversely from what happens in disease spreading, each agent autonomously decide to adopt/advertise a given content. Our daily experience suggests us that the number of the individual that got involved in the process of innovation’s diffusion and the way in which contents spread depend by several conditions. The structure of the social network individuals are part of as well as their sensitivity to peer pressure and/or to media advertising campaigns are examples of constraints that deeply affect such kind of phenomena.

Although often treated as similar processes, diffusion of information and epidemic spreading can be easily distinguished by a peculiar feature: the degree of *activeness* of the subjects they affect. Indeed, the spreading process of a virus does not require an *active* participation of the individuals that catch it (i.e., even though some behavior acts as contagion facilitators – scarce hygiene, moist and crowded environment – we can assume that no one chooses to get the flu on purpose); conversely, we can argue that the diffusion of an idea, an innovation, or a behavior strictly depends not only on the social pressure but also on individual choices.

Such context dependent dichotomy leads to our problem definition:

### **Definition 1**

(Active-Passive Conundrum) Given a **social context** – described as a graph *G*=(*V*,*E*), where a node *v*∈*V* is an individual and an edge (*u*,*v*)∈*E* identifies a social tie among *u*,*v*∈*V* – a **content*ψ*** and a set of adopters $I_{t_{0}}\subset V$ of *ψ*: how can be modeled, and what characterize, *passive* and *active* diffusion processes of *ψ* over *G*?

To address diffusive phenomena related to the typology of content we are interested in this work are often adopted variants of the *Threshold Model* in which adoptions performed by individuals are subject to personal thresholds (identified as the peer pressure exercised by each friend or, theoretically drawn by a given distribution). We can argue that these approaches are only able to capture the *passive* component of the diffusion since they ignore the user interest concerning the content *ψ*, thus assimilating information diffusion to a special case of biological contagion.

To address the active-passive conundrum, in the following we introduce three conceptual scenarios that will act as guidelines for designing and comparing different diffusion models applicable to our specific context.

### Passive, active and mixed scenarios

Since we aim to compare alternative modeling choices able to simulate both *passive* and/or *active* diffusion processes we first need to characterize the scenarios such approaches should describe.

**S1: Passive diffusion.** This scenario assumes that a generic diffusion process takes place independently on the willingness of the individuals. Diffusion relies only on *Peer Pressure*: the more an individual is exposed to a given content the more likely it will adopt it. In such settings, social contagion acts like the virus spreading since, once a sufficient peer pressure is reached the spreading content will affect a target user, leaving no alternatives to individual preferences.

**S2: Active diffusion.** Conversely from the passive diffusion scenario, active spreading assumes that the diffusion process is only *apparent*; each node decides to adopt or not a given content - once known its existence from a peer - only by its interests, completely ruling out peer pressure. Diffusion will then rely only on the adopter *Preference*.

**S3: Mixed diffusion.** This scenario combines *passive* and *active* processes so to shape content diffusion as a mix of the two. In such settings, we assume that individual’s *interests* act as a preferential schema for adoptions but, at the same time, do not neglect the role of *peer-pressure* mechanisms. Novel contents are evaluated by individual preference only when a sufficient peer pressure (exposition) is reached.

Indeed the identified scenarios represent macro-categories that can be studied by applying very different algorithmic models. Moreover, all the models that implement such scenarios are also subject to other *dynamics* peculiar of social contexts, i.e., (i) *spontaneous* adoptions and (ii) *evolution* of the social tissue.

In real-world contexts, individuals can adopt a given content even without the need of being exposed to it through their social circle. All those adoptions that are not endogenous w.r.t. the observed social structure can be considered *spontaneous* (even though they can also be ascribed as results of exogenous phenomena not captured by the modeled system, e.g., news channels, advertising, online/offline media). Spontaneous adoptions can act as diffusion linchpins since their presence, or absence, deeply affect the unfolding of diffusive phenomena.

In the following, we will detail the algorithmic choices made to provide simulations of the sketched scenarios considering the presence/absence of spontaneous adoptions and the presence/absence of blocked nodes.

### Diffusion models

To understand the differences between the proposed scenarios we simulate them with the following diffusion models:

**S1: Threshold model.** We employ the classic *Threshold Model* (Granovetter 1978) to simulate a *passive adoption* process by using a theoretical distribution for the adoption threshold as done in Watts (2002). In the Threshold model during an epidemic, a node has two distinct and mutually exclusive behavioral alternatives, e.g., it can adopt or not the spread content. The *decision* to adopt depends only on the percentage of node’s neighbors that have already adopted the content. As shown in Algorithm 1, the model works as follows:


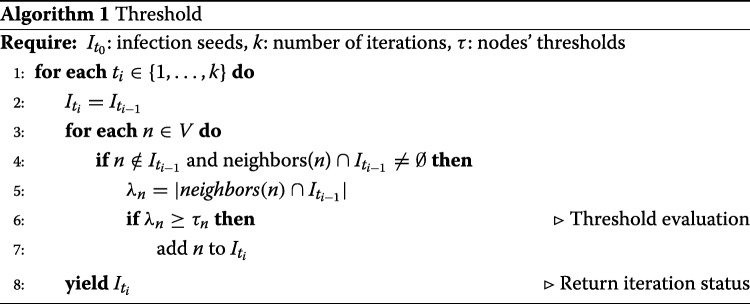

- Each node starts with its own threshold *τ* and status (infected or susceptible);
- During each iteration *t*_*i*_∈*T*, every node *n*∈*V* is observed: iff the percentage of *n* neighbors that were infected until time *t*−1 is greater than its threshold *τ*_*n*_, *n* becomes infected as well.

**S2: Node Profile model**. We design a novel model, called *Node Profile*, to simulate *active* adoptions. In such model, each adopter chooses to adopt the given content based only on his personal preferences. Each node carries its *profile* *γ* describing the degree by which it is likely to accept a content similar to the one that is currently spreading. The diffusion process starts from a set of nodes that have already adopted the content *ψ*. For each of the susceptible nodes in the neighborhood of a node *n* that has already adopted *ψ*, a random value *v* in [0,1] is extracted; if *v*≥*γ*_*n*_ the node adopts the content, otherwise the node refuses to adopt. Susceptible nodes are allowed to change their opinions during every iteration. We also implemented a variant of such model which contemplates blocked nodes, e.g., nodes that after having refused the adoption, with probability *p* decide to stick with their choices permanently. The pseudo-code for the introduced approach is described in Algorithm 2.


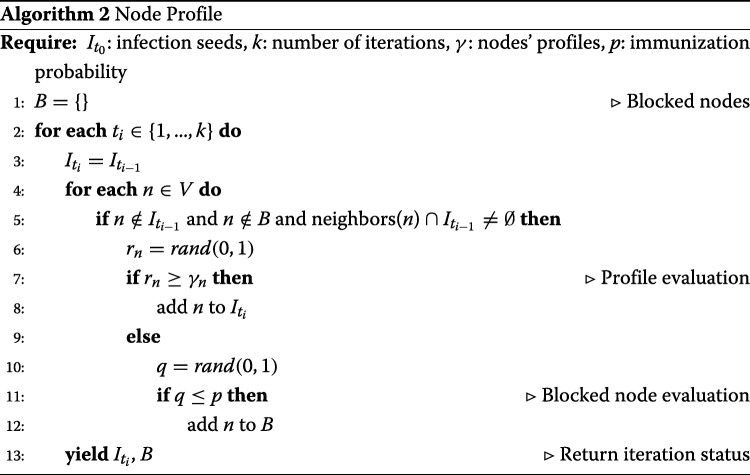


**S3: Profile-Threshold model.** To support mixed behaviors we implement a *Node Profile-Threshold* model that combines the previously described Node Profile model with the peer pressure information (i.e., classic threshold model). This model firstly evaluates if the peer pressure a node receives is enough to overcome its threshold, then if such a constraint is satisfied, it evaluates the node profile. As for the Node Profile model, we implemented a variant that contemplates blocked nodes. Pseudocode for Profile-Threshold model is shown in Algorithm 3.


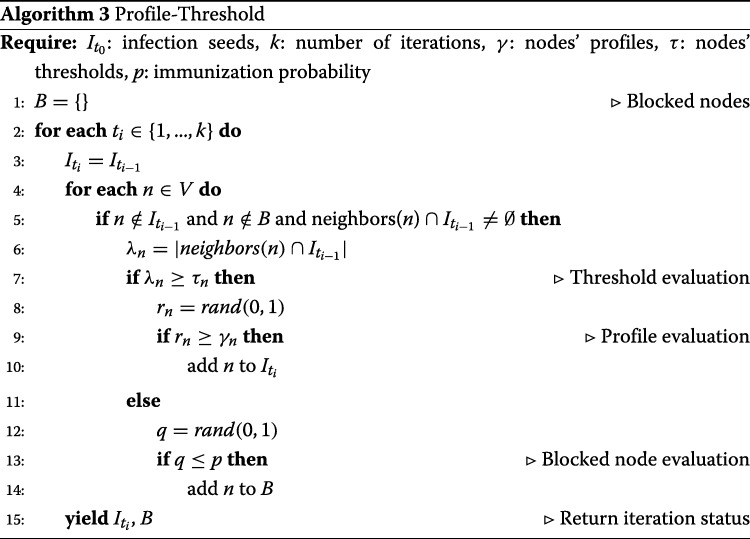


To model spontaneous adoptions we introduced, as the first step before each simulation iteration, a node-wise stochastic process that with a fixed probability *p* transform a susceptible node into an infected one.

All the described diffusion models, have been implemented and made available within the python library *“NDlib”*^1^(Rossetti et al. 2017; Rossetti et al. 2018).

## Experimental analysis

To compare the impacts of active and passive scenarios, we carried out a data-driven investigation modeling the social graph with both synthetic networks as well as with real-world network datasets, as described in Datasets. The analytical protocol we adopted is described in Analytical protocol and the evaluation in Analysis.

### Datasets

For our simulations, we use a real-world dataset, the FB network. This is a sample of the WOSN2009 (Viswanath et al. 2009) dataset and describes online interactions between Facebook users. The FB graph is composed of 31 daily snapshots covering the month of January 2007: statistics of the graph are reported in Table 1. We conduct our experimentation analysis on the static scenario, so we collapsed all FB snapshot graphs in a single network composed by the union of individual node and edge sets.

**Table 1:** Base statistics of the analyzed Facebook graphs

Nodes	Interactions	Edges	CC	*#*Observation	Average degree
63 392	304 392	816 886	13	365 days	13

*CC* identifies the number of connected components

Moreover we simulate the introduced diffusion models also on three synthetic network generator models: (i) Barabási-Albert (1999), (ii) Erdós-Renyi (1959) and (iii) Wats-Strogatz (1998). To have “comparable networks” to the real one, we fix the number of nodes and the average degree such as the characteristics reported in Table 1. So the generated networks have 63392 nodes each, and are obtained by setting the following parameter values:

- Barabási-Albert graph: number of connections per new node *m*=13;
- Erdós-Renyi graph: edge creation probability *p*=0.0004;
- Wats-Strogatz graph: node neighbors *k*=13, rewiring probability *p*=0.01.

### Analytical protocol

To compare the diffusion scenarios previously described, we designed the following analytical protocol:

- For each dataset we randomly selected 100 sets of nodes each one covering 5% of *V*: such sets identify, for each scenario and model, 100 different initial seeds of infection configuration – $I_{t_{0}}$;
- For each dataset, scenario and $I_{t_{0}}$ we executed the active, passive and mixed diffusion models previously introduced for an equal, fixed, number of iterations (30 for all the networks);
- Finally, we compared the models by analyzing the obtained infection trends as well as the percentage of infected nodes at the end of each simulation.

To mitigate the effects of initial seed set selection, we considered as infection trend for each configuration the iteration wise average of the runs over the executions performed while varying the seeds. The same strategy is also applied to identify the final percentage of infected nodes at the end of each configuration simulation.

Finally, to understand the impact of different values of model parameters have on the diffusion process, we simulated the three scenarios with several configurations of the node threshold, *τ*, and node profile *γ*. Moreover, we also varied the immunization probability value, *p*, and spontaneous adoption rate, *a*. As a result, we instantiated all the – valid – parameter combinations for the selected models, varying their values in the following ranges:

- Threshold, *τ*: [0.1, 0.2, 0.3, 0.4, 0.5, 0.6, 0.7, 0.8]
- Node Profile, *γ*: [0.05, 0.1, 0.2, 0.3, 0.4, 0.5, 0.6, 0.7, 0.8]
- Percentage of blocked nodes, *p*: [0, 0.1, 0.2, 0.3]
- Probability of spontaneous adoption, *a*: [0, 0.001, 0.005, 0.01]

### Analysis

The typical strategy to resolve this diffusion problem is to use a Threshold model (a *passive* approach). But the question is: “a *passive* approach is the right way to resolve the diffusion of information problem?”.

To answer this question, in the following, we report the diffusion trends obtained after our simulations for all the networks in a simple scenario – without immunization and spontaneous adoptions. All other scenarios (in which *p*≠0 and/or *a*≠0) are detailed only for the Facebook graph.

#### Results

To better characterize the obtained results we analyze separately models that contemplate blocked nodes from the ones that do not. We treat similarly the results obtained in presence/absence of spontaneous adoptions.

**Without immunization, without spontaneous adoptions.** In this scenario fall the standard implementation of the three methods; we analyze separately networks to better characterize the differences between the methods.

**Fig. 1:**
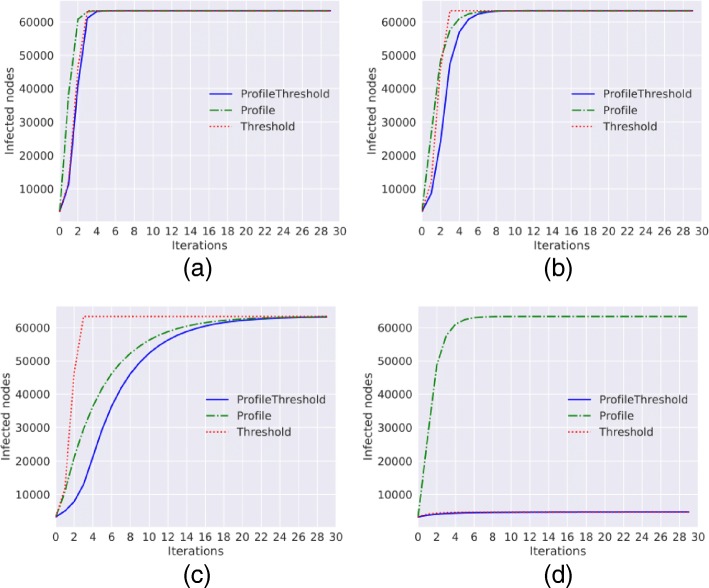
Diffusion trends for the Barabasi Alberth graph. Diffusion trends for *Threshold*, *ProfileThreshold* and *Profile* model with *a*=0 and *p*=0 and with different values of *γ* and *τ*. **a** *γ*=0.1,*τ*=0.1**b** *γ*=0.4,*τ*=0.1**c** *γ*=0.8,*τ*=0.1**d** *γ*=0.4,*τ*=0.2

**Barabasi Albert graph.** The diffusion trends obtained with the simulation of the three methods on the Barabasi Albert graph are shown in Fig. 1.

As we can observe, if we fix the threshold (*τ*) equal to 0.1, with low values for the Node Profile (*γ*) the diffusion trends obtained with the three methods are very similar (Fig. 1a, b). The three models show a fast grow; after only four iterations almost all the nodes of the network are infected. If we change the value of *γ*, and we fix it to 0.8, as shown in Fig. 1c the growth of the number of infected nodes obtained with the Profile model is slower compared to the previous figures; only at the end of the observation period, the trend reaches the total number of infected nodes. This result shows that the peer pressure is significant; even if the people do not like the content spread (every people has the 20% of percentage to accept a content similar to the one that is currently spreading), they end up adopting it. With only a threshold equal 0.2 the spread does not start; in average, each node has 13 neighbors and with the choice of threshold equal 0.2 the node can become infected after three infected neighbors (Fig. 1d).

**Fig. 2:**
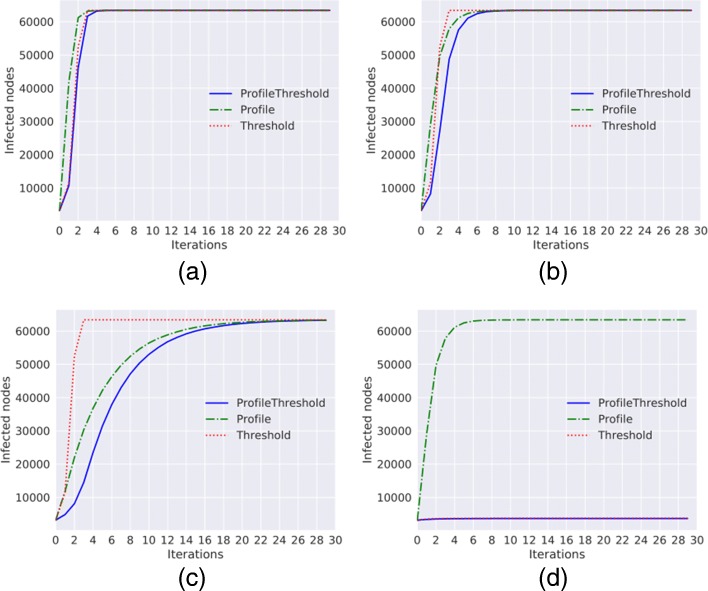
Diffusion trends for the Erdós-Renyi graph. Diffusion trends for *Threshold*, *ProfileThreshold* and *Profile* model with *a*=0 and *p*=0 and with different values of *γ* and *τ*. **a** *γ*=0.1,*τ*=0.1**b** *γ*=0.4,*τ*=0.1**c** *γ*=0.8,*τ*=0.1**d** *γ*=0.4,*τ*=0.2

**Erdós-Renyi graph.** As we can see from the Fig. 2 the behavior of the diffusion trend for the Erdós-Renyi graph is very similar to the results obtained with the Barabasi Alberth graph.

**Fig. 3:**
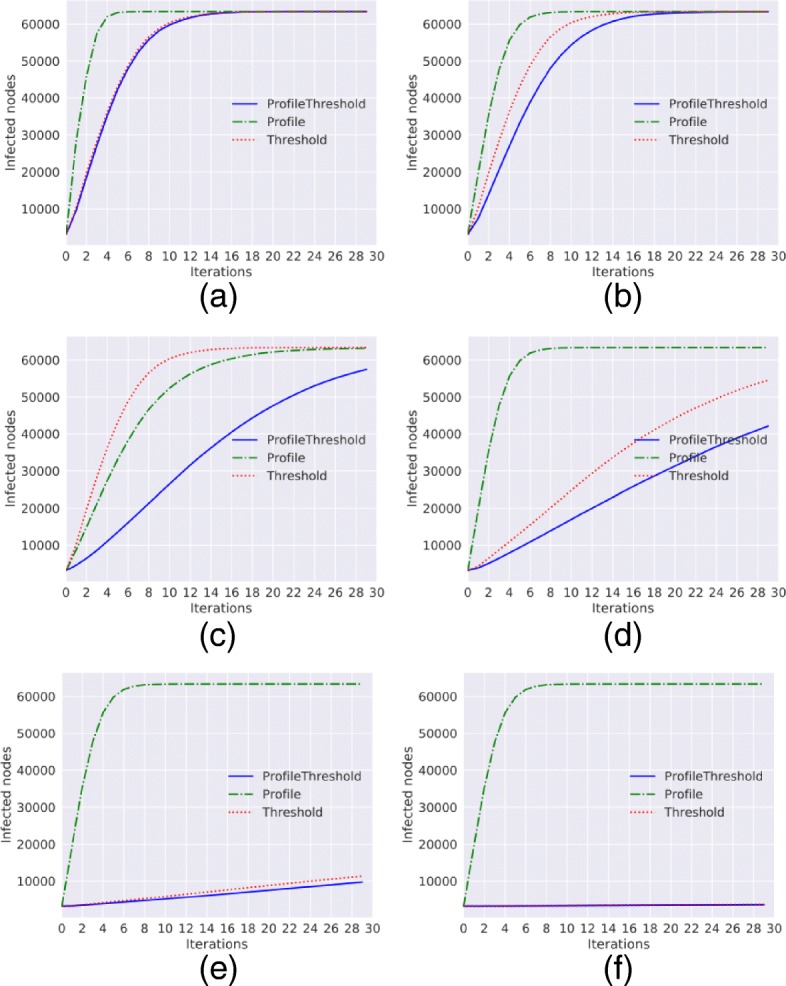
Diffusion trends for the Wats-Strogatz graph. Diffusion trends for *Threshold*, *ProfileThreshold* and *Profile* model with *a*=0 and *p*=0 and with different values of *γ* and *τ*. **a** *γ*=0.1,*τ*=0.1**b** *γ*=0.4,*τ*=0.1**c** *γ*=0.8,*τ*=0.1**d** *γ*=0.4,*τ*=0.2**e** *γ*=0.4,*τ*=0.3**f** *γ*=0.4,*τ*=0.4

**Wats-Strogatz graph.** The diffusion trends for the Wats-Strogatz graph are shown in Fig. 3. For this network, the diffusion process is slower compared to the other networks; in this case, we do not have particular nodes, such as hubs that speed up the diffusion, nor an evident small-world effect (due to the chosen parameter values). In fact, we fix *p*=0.01 and the network is more similar to a lattice than a random network, so the results of the three diffusion models are little dependent from the initial infected node sets $I_{t_{0}}$. For this network, differently from the previous graphs, with a value of threshold equal 0.2, the diffusion process starts. As shown in Fig. 3d, even if the number of infected nodes is less than the number obtained with the Profile model (with *γ*=0.4) the two models with the threshold reach around the 70% of infected nodes at the end of the process. Conversely, with a threshold equal to 0.3 only the 16% of node became infected and with *τ*=0.4 the diffusion process does not start.

**Fig. 4:**
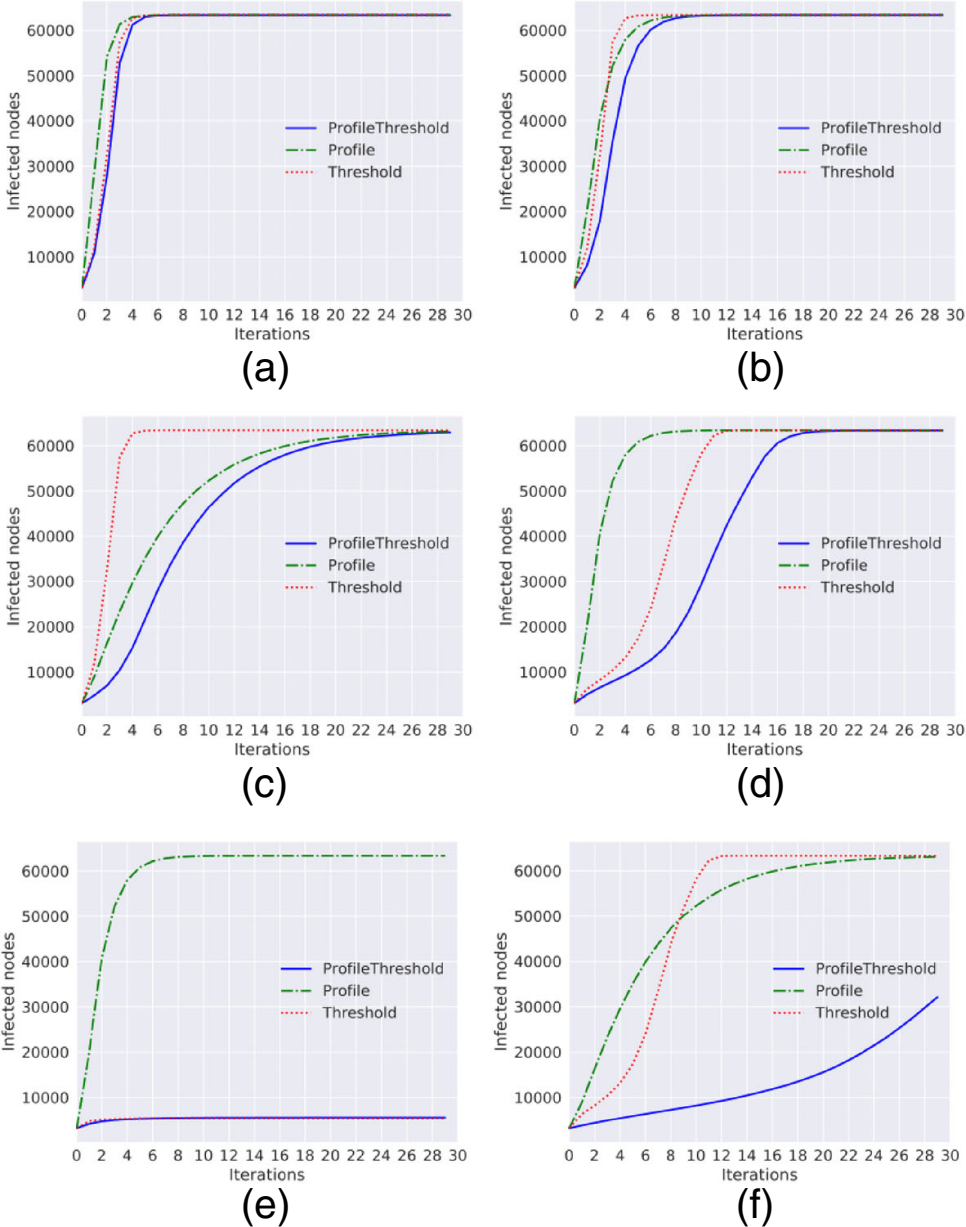
Diffusion trends for the Facebook graph. Diffusion trends for *Threshold*, *ProfileThreshold* and *Profile* model with *a*=0 and *p*=0 and with different values of *γ* and *τ*. **a** *γ*=0.1,*τ*=0.1**b** *γ*=0.4,*τ*=0.1**c** *γ*=0.8,*τ*=0.1**d** *γ*=0.4,*τ*=0.2**e** *γ*=0.4,*τ*=0.3**f** *γ*=0.8,*τ*=0.2

**Facebook graph.** Also for the real network, we obtain results similar to the synthetic networks; with low values for *γ* and *τ* the trends of the three methods are very similar as we can see in the Fig. 4.

For the real network and for the Wats-Strogatz graph we obtain an expected result: the *active* diffusion trends show the fastest growth; conversely, the *passive* diffusion trends seem to be tied to a slower start. Such results are somehow expected: the former model assumes that a susceptible node can decide to adopt when it discovers the existence of a given information (e.g., when at least a single of its neighbors has already adopted it) while the latter fixes an exposure threshold below which the node does not come in contact with the information. Particular attention should be reserved to the *mixed* approach, described by the Profile-Threshold model: for the first two synthetic networks, the mixed and passive models behave alike while in the Facebook and Wats-Strogatz network the Profile-Threshold trend stands below the Threshold one.

This result is also expected: with that approach, first, a susceptible node has to overcome the exposure threshold to come in contact with the information, after, he has to decide to adopt it. So to adopt the information, two conditions are necessary: (i) the node has to have a sufficient number of adopted neighbor and (ii) he has to autonomously decide to adopt the information because he loves it.

**Fig. 5:**
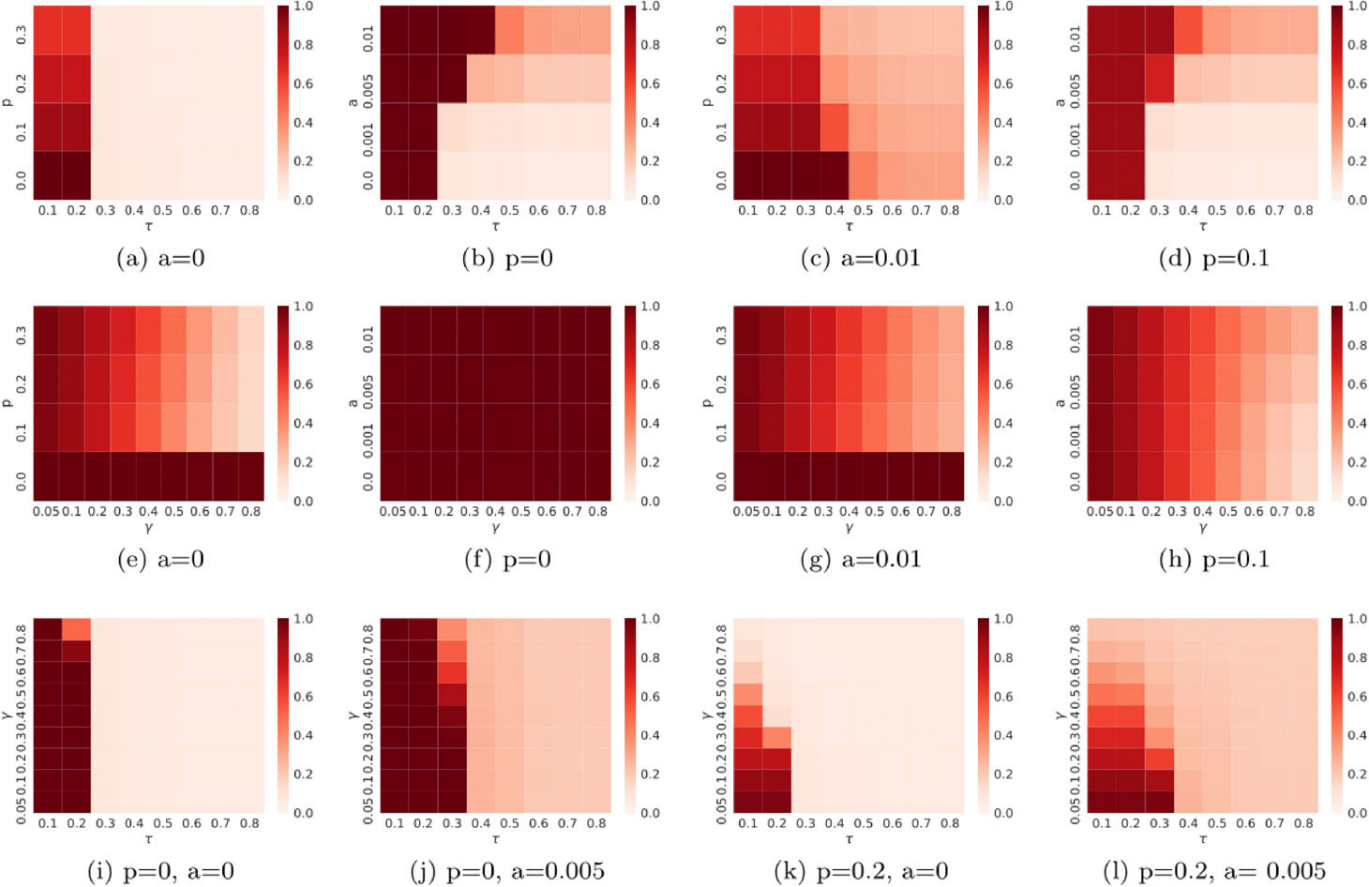
Heatmap for the Facebook graph. Heatmap for the Facebook network of the percentage of infected nodes at the end of the observed period in the static case. In the first row the results obtained with the Threshold model; in the x-axis we have the *τ* parameter and in the y-axis the spontaneous adoption rate *a*, for (**b**) and (**d**) and the immunization probability *p* for (**a**) and (**c**). In the center row the results obtained with the Profile model; in the x-axis we have the *γ* parameter and in the y-axis the spontaneous adoption rate *a*, for (**f**) and (**h**) and the immunization probability *p* for (**e**) and (**g**).Finally, in the bottom row the results obtained with the ProfileThreshold model; in the x-axis we have the *τ* parameter and in the y-axis the *γ* parameter. In this case, for each heatmap we fix at the same time the values of spontaneous adoption rate *a* and for the immunization probability *p* as reported in the captions (**i**–**l**)

**Without immunization, with spontaneous adoptions.** For this scenario we show in Fig. 5 the heatmap obtained with the three methods on the real network Facebook. Every cell of the heatmap represents the percentage of infected nodes at the end of the observed period (in our case at the end of the 31st day) for different parameters. The cells with a darker shade of red have a percentage of infected node high; the cells with a lighter shade of red have a low percentage. We expected that with the introduction of the spontaneous adoptions the percentage of infected nodes increase. We can observe this result for all the methods.

For the Threshold model (Fig. 5b) in the x-axis we have the *τ* parameter and in the y-axis the spontaneous adoption rate *a*. On the top of the heatmap, the percentage of infected nodes is greater compared to the bottom, where the value of *a* is small (the range of *a* is from 0 to 0.01). For the Profile model (Fig. 5f), differently from the previous case, in the x-axis we put the *γ* parameter. In this case with each value of *γ* and with *p*=0 the percentage of infected nodes is high; as observed previously with the diffusion trend, the value of the threshold impact mainly the diffusion process. If the percentage of neighbors is below the fixed threshold, the node does not come into contact with the information; he can adopt the idea only spontaneously. This result can be observed also in the Fig. 5i: if *γ* has a big value (i.e. ≥0.4) the diffusion process can not start; this phenomenon is mitigated from the introduction of adopter spontaneous (Fig. 5j).

Also in this case, we find the previous result: the *active* diffusion trends show the fastest growth after we have the trend obtained with *passive* approach and finally the *mixed* approach.

**With immunization, without spontaneous adoptions.** When we introduce the concept of “blocked nodes” the diffusion patterns change. In this case, we want to simulate a random immunization; the nodes that will become immune are pick up at random. As expected the percentage of infected nodes of the three models experience dumping compared to that observed in the previous analysis. In particular, especially for the Profile model (Fig. 5e), we can observe that after the same period the percentage of infected nodes halves compared to the simulation with *p*=0 (the row at the bottom of the heat map). Indeed, our experiments underline a linear correlation between the value chosen for *p* and the observed reduction of the infected population.

**With immunization, with spontaneous adoptions.** When we introduce the concept of “blocked nodes” and “spontaneous adoption” the diffusion pattern that we obtain are those expected. With the increase of the *a* value, the percentage of infected nodes grows; conversely with the increase of the *p* value the percentage decrease, as we can observe in Fig. 5c, d, g, h, k, l.

## Conclusion

In this work we tackled the problem of *activeness* of diffusion phenomena describing different scenarios.

So far, both epidemic spreading and information diffusion have been studied using a common modeling framework. Among the models defined to simulate diffusive processes, we focused our attention on the Threshold one, aiming to describe the diffusion of specific classes of contents: innovations, ideas, behaviors.

Differently from compartmental models (e.g., SI, SIR, SIS) the Threshold model once given an initial infection status produces a deterministic evolution of the diffusion: the lack of a stochastic component, along with the model rationale, makes the diffusion produced by the observed model *passive*, i.e., a process during which the nodes involved do not play any active role. We pointed out how such approach is able only to capture one of the components that regulate the diffusion of contents in a social context (e.g., *peer-pressure*), giving no credit to another important component: *individual preferences*. Moreover, we underlined that such limitation, although acceptable when dealing with disease spreading, deeply simplifies the processes that regulate diffusion in our specific settings.

To cope with such limitation, we designed two stochastic models that reintroduce an *active* role for the nodes: namely, Profile and Profile-Threshold. In our experimentation, we showed how *passive* and *active* strategies impact both the speed and overall width of the diffusion process. Moreover, we underline the need for a *mixed* approach that better simulate the real mechanics of information spreading, modeling both the effect of peer-pressure and individual interest in the content spread.

Moreover, we also analyzed how the presence of spontaneous adopters (i.e., individuals that become “infected” due to exogenous factors) as well as extremists (i.e., individuals who categorically refuse to adopt/change their mind on a given content) can speed up/dampen the diffusion process.

As future work, since social networks are constantly evolving realities where individuals, as well as interactions among them, rise and fall, we plan to reformulate our approaches for dynamic topologies. We plan also to extend our modeling framework to understand better the implications that diffusion processes have on network topology. We will aim to propose a comprehensive double feedback loop system in which the two kinds of evolutive patterns (on and of network) are considered from a holistic perspective, thus allowing to understand better and characterize complex network phenomena. Finally we plan to extend the study of our methods when we introduce the targeted immunization; in fact, the random immunization of nodes has been shown incapable of protecting the population when the contacts distribution is wide.

## Footnotes

1 NDlib: https://github.com/GiulioRossetti/ndlib
